# Supplementary material for: What mechanism design helps to realize the innovation function of maker-spaces: A qualitative comparative analysis based on fuzzy sets
Source: PLoS One. 2022 Sep 9;17(9):e0274307. doi: 10.1371/journal.pone.0274307 (PMC9462774; doi:10.1371/journal.pone.0274307)
Supplement: Attachment — Submitted filename: Review Comments for PONE-D-21-37616.docx [file pone.0274307.s002.docx]

**Review comments** PONE-D-21-37616

Thank you for giving me the opportunity to read this exciting study! This manuscript titled “What mechanism design helps to realize the innovation function of maker-spaces: A qualitative comparative analysis based on fuzzy sets” uses the method of fuzzy set qualitative comparative analysis (fsQCA) to analyze data from 63 maker-spaces in Zhejiang Province and to study proposes a reasonable mechanism design scheme for maker-space. Given the unique research design, the rareness of the maker-spaces data, and robust results, there is no doubt that it is an important paper and an interesting story to read. My comments are as follows:

**Major comments:**

1. This manuscript has a rigorous for the methodology and research analysis section. I still have some concern on part of it. In line 315-316, and line 318-321, the authors mentioned that they use the raw continuous data, binary data, and level data. Since the sample size is small, it would be great if the author can conduct some robustness analysis to make the results more robust. For example, for the continuous result data, taking logarithm would make the estimate more tolerate to outliers.
2. In the robustness test section, it would be great if the author could add a table here or in the appendix to show the robustness check results.

**Minor comments:**

3. This manuscript has a rigorous contribution to the literature by conducting surveys with 63 valid questionnaires. If allowed, a larger sample size would contribute to a more robustness results.
4. It’s nice that the author also discussed the future research and limitations at the end of the paper. It would be helpful if the limitation is discussed in more details.
5. It might be helpful for readers to clearly understand the results if the figure for the results in the main tables is added.

**Comments to editors:**

The overall quality of the manuscript is good. I suggest publishing this paper if the authors could address the issues mentioned above.
